# Hysteretic behavior of bladder afferent neurons in response to changes in bladder pressure

**DOI:** 10.1186/s12868-016-0292-5

**Published:** 2016-08-12

**Authors:** Shani E. Ross, Zachariah J. Sperry, Colin M. Mahar, Tim M. Bruns

**Affiliations:** 1 Department of Biomedical Engineering, University of Michigan, Ann Arbor, MI USA; 2 Department of Nuclear Engineering and Radiological Sciences, University of Michigan, Ann Arbor, MI USA; 3 Biointerfaces Institute, University of Michigan, Ann Arbor, MI USA; 4 NCRC-B20-104W, 2800 Plymouth Road, Ann Arbor, MI 48109 USA; 5 NCRC-B20-111WD, 2800 Plymouth Road, Ann Arbor, MI 48109 USA; 6 NCRC-B10-A169, 2800 Plymouth Road, Ann Arbor, MI 48109 USA

**Keywords:** Hysteresis, Dorsal root ganglia, Bladder afferents, Pelvic nerve, Urinary tract, Bladder, Bladder pressure

## Abstract

**Background:**

Mechanosensitive afferents innervating the bladder increase their firing rate as the bladder fills and pressure rises. However, the relationship between afferent firing rates and intravesical pressure is not a simple linear one. Firing rate responses to pressure can differ depending on prior activity, demonstrating hysteresis in the system. Though this hysteresis has been commented on in published literature, it has not been quantified.

**Results:**

Sixty-six bladder afferents recorded from sacral dorsal root ganglia in five alpha-chloralose anesthetized felines were identified based on their characteristic responses to pressure (correlation coefficient ≥ 0.2) during saline infusion (2 ml/min). For saline infusion trials, we calculated a maximum hysteresis ratio between the firing rate difference at each pressure and the overall firing rate range (or Hmax) of 0.86 ± 0.09 (mean ± standard deviation) and mean hysteresis ratio (or Hmean) of 0.52 ± 0.13 (n = 46 afferents). For isovolumetric trials in two experiments (n = 33 afferents) Hmax was 0.72 ± 0.14 and Hmean was 0.40 ± 0.14.

**Conclusions:**

A comprehensive state model that integrates these hysteresis parameters to determine the bladder state may improve upon existing neuroprostheses for bladder control.

## Background

The lower urinary tract (LUT) has two main functions: storage (continence) and voiding (micturition) of urine. Normal operation of these two functions involves coordinated autonomic and voluntary neural control utilizing local, spinal, and supraspinal pathways [1] and can be affected in many conditions including spinal cord injury, Parkinson’s disease, and multiple sclerosis. A thorough understanding of the LUT physiology can aid in developing better treatments for patients suffering from bladder dysfunction.

Sensory information from the LUT is transmitted to the spinal cord and brain via afferent neurons in the pelvic, hypogastric, and pudendal nerves [2]. Afferent information from the bladder is primarily transmitted by pelvic nerves that originate in the caudal lumbosacral dorsal root ganglia (DRG) [3, 4]. These afferents are mainly divided into myelinated Aδ-fibers and unmyelinated C-fibers. Aδ-fibers are mechanosensitive and respond primarily to passive bladder distension and active contractions [5–7]. These fibers typically encode bladder wall tension and/or strain [8, 9] and bladder pressure [6, 10, 11] and are considered to be tension receptors ‘in-series’ with the muscle fibers of the bladder wall [12]. Some mechanosensitive fibers respond to bladder distension but not contractions and are thought to instead encode volume, irrespective of pressure [13], with receptors “in-parallel” with the muscle fibers [14]. C-fibers, in contrast, generally respond to noxious stimuli such as chemical irritants, cold infusants, and high pressures (greater than 30 mmHg) [14, 15], but have also been reported to respond to volume [13].

Mechano-sensitive Aδ-fibers are silent when the bladder is empty, but begin to fire once a pressure threshold is reached and gradually increase their firing as the bladder fills and pressure rises [6, 10, 12, 16, 17]. Pressure thresholds for these neurons are typically between 5 and 20 cmH_2_0 [3, 10, 17, 18], with neurons of different pressure thresholds being successively recruited as the bladder fills [16]. Some of these fibers decrease their firing or plateau at high pressures [14]. However, the relationship between bladder afferent activity and intravesical pressure is not a simple linear one. Firing rate responses to pressure can differ depending on prior activity, demonstrating hysteresis in the system [8, 16].

Hysteresis is a nonlinear phenomenon in which the output of a system depends on both the current input and recent history. It commonly occurs in ferromagnetic and ferroelectric material but is present in other systems including many mechanosenory systems [19], for example, in stretch receptors in cray fish [20] and muscle spindles in cats [21]. Hysteresis is also a property of smooth muscle, which makes up the detrusor layer in the bladder wall [22].

Hysteresis in the bladder pressure-bladder afferent relationship has been highlighted in previous LUT literature [8, 16, 23] but has not been quantified or compared across different bladder states. The goal of this study was to quantify the hysteretic relationship between bladder afferent activity and bladder pressure during non-micturition bladder contractions as the bladder is being filled and also when it is at an isovolumetric state. Neural activity was recorded from sacral dorsal root ganglia at the S1 and S2 levels, which contain the cell bodies of pelvic neurons, in cats. Neurons that responded to bladder filling demonstrated a quantifiable hysteresis that was similar to examples found in the published literature. A better understanding of this hysteretic relationship could be utilized to implement a comprehensive state model for a closed-loop bladder neuroprosthesis, in which pressure is estimated from afferent activity and stimulation is delivered for bladder control accordingly. Such a system could be used clinically for patients suffering from spinal cord injury and other neurogenic bladder disorders.

## Methods

### Subjects

Five intact adult male cats (age: 0.9–1.4 years old, 4.2–5.2 kg, domestic short-haired, Liberty Research, Inc, Waverly, NY) were used in non-survival experiments in this study with one cat used per experiment. All procedures were approved by the University of Michigan Institutional Animal Care and Use Committee, in accordance with the National Institute of Health’s guidelines for the care and use of laboratory animals.

### Experimental setup and surgical procedure

Animals were initially anesthetized with a ketamine (6.6 mg/kg)–butorphanol (0.66 mg/kg)-dexmedetomidine (0.033 mg/kg) intramuscular (IM) dose, intubated, and then maintained on isoflurane anesthesia (2–4 %) during surgical procedures. Respiratory rate, heart rate, end-tidal CO_2_, O_2_ perfusion, temperature, and intra-arterial blood pressure were monitored continuously using a Surgivet vitals monitor (Smiths Medical, Dublin, OH). Intravenous (IV) lines were inserted into one or both cephalic veins for drug and fluid infusions. Intravenous fluids (1:1 ratio of lactated Ringers solution and 5 % dextrose) were infused at a rate of 5–10 ml/kg/h and increased up to 30 ml/kg/h during surgery as needed.

A catheter was inserted into the bladder for intravesical fluid infusion and pressure monitoring. In experiments 1, 3, and 4, an abdominal midline incision was performed to expose the bladder and a 6 Fr supra-pubic dual-lumen catheter (Laborie, Williston, VT) was inserted into the dome of the bladder and secured with a purse-string suture. A single-lumen 3.5 Fr catheter (Utah Medical Products, Midvale, UT) and dual-lumen 3.5 Fr catheter was inserted into the bladder via the urethra in experiments 2 and 5, respectively.

Following bladder line placement, a midline dorsal cut was made to expose vertebrae from L7 to S3. The spinal muscles were reflected from the vertebrae and a laminectomy was performed to access sacral DRG (S1–S2) in the cat [18]. Iridium oxide microelectrode arrays (5 × 10 and 4 × 10 ICS-96 MultiPort split planar arrays, Blackrock Microsystems, Salt Lake City, UT) were implanted into the DRG either bilaterally or unilaterally using a pneumatic inserter (Blackrock Microsystems). These types of multielectrode arrays are a standard approach for identifying and recording from dozens of neurons simultaneously in DRG [18, 23–27] and peripheral nerves [28–30]. For experiments 1 and 5 the 5 × 10 array was inserted in the left S1 DRG and the 4 × 10 into the left S2 DRG. For experiments 2, 3, and 4, 5 × 10 arrays were inserted bilaterally in S1 and 4 × 10 arrays were inserted bilaterally in S2. Microelectrode shank lengths were either 0.5 or 1.0 mm with 0.4 mm inter-shank spacing. The ground wires were connected to a stainless steel needle inserted in the skin lateral and caudal to the laminectomy incision site and reference wires were placed near the spinal cord. Animals were then transitioned to alpha-choloralose (70 mg/kg induction; 20 mg/kg maintenance; doses given IV) for subsequent testing. Alpha-chloralose anesthesia was augmented with buprenorphine (0.01 mg/kg; given every 8–12 h IV).

**Fig. 1:**
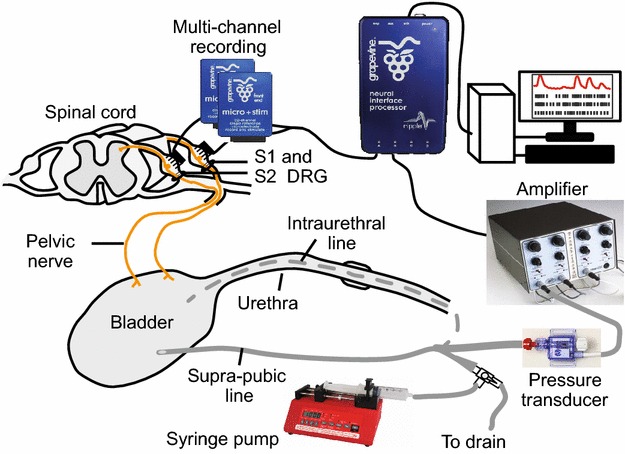
Experimental setup. Illustration of a cross-section through the spinal cord highlighting the pelvic nerves and dorsal root ganglia at the sacral level. *Arrays* were implanted in S1 and S2 DRG. Saline was infused into bladder either via a supra-pubic line or an intraurethal line. Intravesical pressure was monitored with a pressure transducer and amplifier. Both neural data and pressure were recorded with a Grapevine data acquisition system. Image modified from Bruns et al. [57]

DRG neural data was acquired at a rate of 30 kHz and band-passed filtered (250 Hz–7.5 kHz) using a Grapevine neural interface processer and Trellis recording system (Ripple, Salt Lake City, UT). A global amplitude threshold, between −20 and −35 µV (depending on the noise amplitude), was set for all electrode channels. Any signal crossing of the threshold was captured as a spike snippet and stored for offline analysis. Bladder pressure was monitored using a pressure transducer (DPT-100, Utah Medical Products, Midvale, UT) and transducer amplifier (World Precision Instruments, Sarasota, FL). The bladder pressure signal was recorded with the Grapevine system at 1 kHz. During testing (see "Experimental procedures" section) saline was infused into the bladder using a syringe pump (New Era Pump Systems, Inc., Farmingdale, NY). Figure 1 shows the experimental set-up.

### Experimental procedures

#### Slow fill trials

The bladder was first emptied using the bladder catheter. Sacral DRG neural activity was recorded while saline was infused into the bladder at a near physiological rate of 2 ml/min [31]. In most trials, this was done until dripping from the external meatus or around the urethral catheter (for those experiments where the catheter was inserted via the urethra) was observed. This infused volume was defined as the “leak volume” for a given experiment. For trials in which infusion was stopped before leaking was observed, then the leak volume from a prior fill sequence was assumed. For experiments 1–4, room-temperature saline (22 °C) was used; whereas for experiment 5, body-temperature saline (41 °C) was used. Two infusion trials per experiment (cat) in which there were only non-voiding bladder contractions were used in the analysis.

#### Isovolumetric trials

In experiments 3 and 4, isovolumetric trials were performed with the bladder volume within 20–50 ml, while assuming negligible urine generation. Neural activity and bladder pressure were recorded for non-voiding bladder contractions.

After completion of all testing, animals were euthanized with a 3 ml intravenous dose of sodium pentobarbital (390 mg/ml) while under deep anesthesia.

### Data analysis

After data collection, spike snippets were sorted in Offline Sorter v3.3.5 (Plexon, Dallas, TX), using principal component analysis followed by manual review to identify unique spike clusters. In MATLAB (Mathworks, Natick, MA), instantaneous firing rates for each unit were calculated at intervals of 0.5 s. Discrete spike events were converted into a smoothed time series of firing rates using a non-causal linear filter with triangular kernel of width 1 s [27]. Bladder pressure was filtered (4 Hz low pass). Units whose firing rates highly correlated with bladder pressure (correlation coefficient, ρ ≥ 0.2) over the course of a saline infusion trial were identified as bladder units. These units were confirmed visually to increase firing with increasing bladder pressure as shown in the literature [6, 10, 16, 18].

**Fig. 2:**
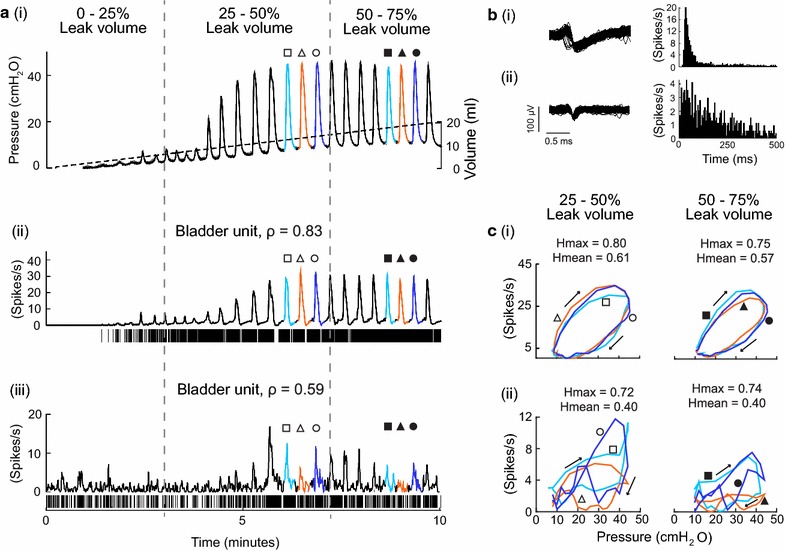
Demonstration of hysteresis in two example units. **a** *i* Bladder pressure trace with periods corresponding to bladder contractions highlighted (*different colors* and *different symbols*) and volume infused (*black dotted line*). *Open symbols* are used to indicate contractions during 25–50 % leak volume and *closed symbols* are used to indicate contractions during 50–75 % leak volume. *ii* Firing rate for a bladder unit that correlates well with pressure during the same time period (*symbols* correspond to those used in *i*, with correlation coefficient (ρ) indicated). *iii* Firing rate for a bladder unit that is weakly correlated with bladder pressure. **b** Waveform and interspike interval (isi) histogram, with bin width = 5 ms, for neurons in **a**. **c** Bladder pressure plotted against firing rate of respective neurons from **a** for each contraction during the designated periods at 25–50 % leak volume (*left column*) and 50–75 % leak volume (*right column*). The Hmax and Hmean values are given above the plots. *Arrows* represent direction of cycle

**Fig. 3:**
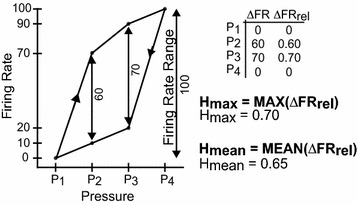
Hysteresis calculation method. Diagram of pressure versus firing rate illustrating how the hysteresis indices, Hmax and Hmean, were calculated. P_i_ corresponds to discrete pressures. The firing rates at the lowest and highest pressure were excluded from the Hmean equation

Hysteresis in the bladder pressure-firing rate relationship was calculated during bladder contractions using a method derived from Kosmulski et al. [32] for electrochemical capacitors. Sets of three contractions (pressure change ≥ 10 cmH_2_O, stratified by 25 % intervals of the leak volume for slow fill trials) were used for hysteresis calculations (Fig. 2a). The start and end of each contraction was determined based on pressure inflection points. For each contraction, the pressure trace was divided into 2 cmH_2_0 bins and the mean firing rate corresponding to pressure within each bin was calculated. The mean firing rate and pressure were plotted against each other (Fig. 2c). Pressure ranged from a minimum value (P_min_) to a maximum value (P_max_) with a corresponding firing rate range from FR_min_ to FR_max_. Figure 3 shows a stylized diagram of pressure plotted against firing rate demonstrating how the hysteresis values are calculated. For each binned pressure value (P_1_, P_2_, P_3_, and P_4_), the difference in firing rate (ΔFR) is calculated and divided by the firing rate range (FR_max_ − FR_min_). This ratio is defined as ΔFR_rel_. The following two hysteresis indices were computed: Hmax and Hmean. Hmax is the maximum ΔFR_rel_ and Hmean is the average ΔFR_rel_. The start and end points were excluded in the Hmean calculation to avoid overrepresentation of the narrow ends of the pressure-firing rate curves. Hmax and Hmean were then averaged across the 3 contractions. Hmax and Hmean are dimensionless values ranging from 0 to 1, where 0 represents no hysteresis and 1 represents maximum hysteresis.

### Statistical analysis

A linear mixed models ANOVA with volume range as the fixed effect and a random intercept of animals (experiments) was carried out to determine if there was any statistical difference in Hmax or Hmean values between volume ranges for both slow fill and isovolumetric trials. Given the small number of animals and variation in the number of bladder units observed per experiment, we went with the linear mixed models with experiment as the random term in the model. *P* values (significance level α < 0.05) were obtained by likelihood ratio tests of the full model with the effect in question (volume) against the model without the effect in question [33]. To test for significance between experiments, an ANOVA linear model was performed. Post hoc analysis was done using the Tukey–Kramer multiple comparison test. Relationships between Hmax and Hmean, Hmax and ρ, and Hmean and ρ were assessed using regression analysis. MATLAB and Microsoft Excel (Redmond, WA) were used to perform statistical tests. Values are reported as mean ± standard deviation.

## Results

### Bladder units

Seventy units from five experiments were identified as bladder units (ρ ≥ 0.2), with 57 units in S1 DRG and 13 units in S2 DRG. These units were quiescent at very low pressures and increased firing as bladder pressure increased. Four of those units did not correlate with bladder contractions and were excluded from further analysis. Forty-six units were present in the slow fill trials with average ρ = 0.57 ± 0.16 (correlation analysis done over the infusion period, n = 5 experiments). Correlation coefficients did not differ significantly across experiments [F(1,44) = 1.65, *p* = 0.21]. For the isovolumetric trials, there were 33 units with average ρ = 0.62 ± 0.19 (correlation computed over the period of analysis, n = 2 experiments). Correlation coefficients did not differ significantly across experiments [F(1,31) = 0.02, *p* = 0.89]. Thirteen units overlapped in both the slow fill and isovolumetric trials.

### Hysteresis for saline infusion and isovolumetric trials

**Fig. 4:**
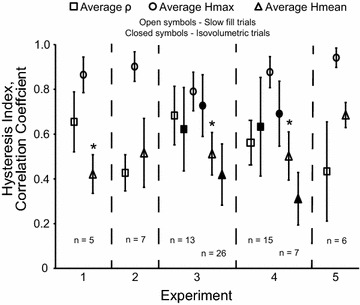
Average correlation coefficient (ρ), Hmax, and Hmean for each experiment. Values are given for the slow fill trials (*open symbols*) and isovolumetric trials (*closed symbols*). The number of bladder afferents used in each experiment are also given. For experiments 3 and 4, the number of bladder afferents used in the slow fill trials are displayed to the *left* of the *column* and the number of bladder afferents used in the isovolumetric trials are given to the *right*. *Error bars* represent standard deviation, *p < 0.05 with respect to Hmean from experiment 5

Figure 4 shows the average Hmax and Hmean indices, along with the average correlation coefficient, for each experiment. The average Hmax was 0.86 ± 0.09 with values ranging from 0.68 to 0.99 and did not differ significantly across experiments [F(1,44) = 1.61, *p* = 0.21]. The average Hmean was 0.52 ± 0.13 with values ranging from 0.27 to 0.76. Hmean was significantly higher in experiment 5 compared to experiments 1, 3, and 4 [F(1,44) = 9.31, *p* < 0.01]. For the isovolumetric dataset, the average Hmax was 0.72 ± 0.14 with values ranging from 0.38 to 1.00 and the average Hmean was 0.40 ± 0.14 with values ranging from 0.09 to 0.73. There was no significant difference in Hmax [F(1,31) = 0.39, *p* = 0.53] or Hmean [F(1,31) = 3.85, *p* = 0.06] among experiments.

**Fig. 5:**
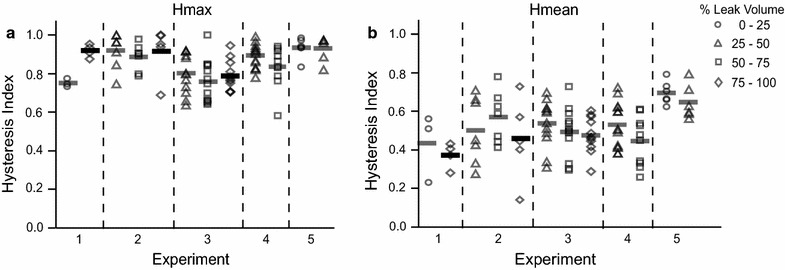
Hysteresis values for slow fill trials. Hmax (**a**) and Hmean (**b**) for each experiment, segregated by volume. Each data point corresponds to a unit. *Different symbols* correspond to Hmax and Hmean calculated for a unit for a particular % leak volume range. *Horizontal bars* are the average Hmean and Hmax value for a given % leak volume interval

**Fig. 6:**
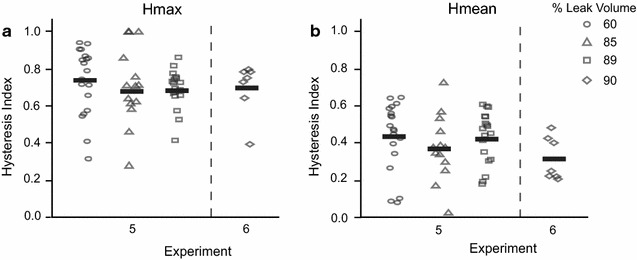
Hysteresis values for isovolumetric trials. Hmax (**a**) and Hmean (**b**) values for each experiment, segregated by volume. Each data point corresponds to a unit. *Different symbols* correspond to Hmax and Hmean calculated for a unit for a particular % leak volume. *Horizontal bars* are the average Hmean and Hmax value for a given % leak volume

For slow fill trials, there was no difference in hysteresis observed at different volume ranges (Hmax: χ^2^ (3) = 5.91, *p* = 0.12; Hmean: χ^2^ (1) = 0.83, *p* = 0.36) (Fig. 5). Note, given the variability in the number of contractions produced for the different animals, some units only had hysteresis values for one given volume range, whereas others had hysteresis values for multiple volume ranges. Figure 6 shows the breakdown of Hmax and Hmean across different volumes for the isovolumetric trials. There was no difference between volumes (Hmax: χ^2^ (1) = 0.70, *p* = 0.40; Hmean: χ^2^ (1) = 3.04, *p* = 0.08).

**Fig. 7:**
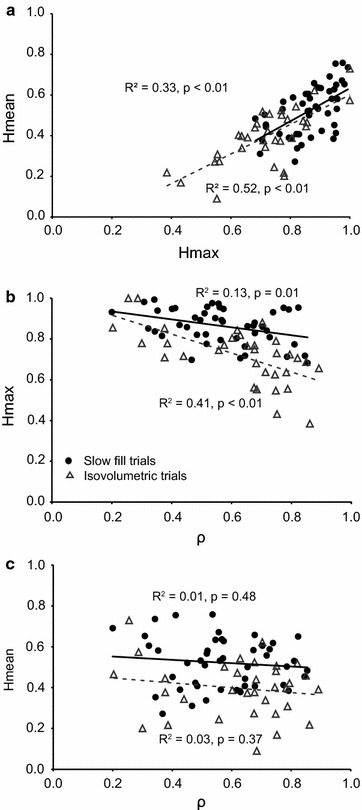
Regression results. **a** Scatter plot of Hmean versus Hmax for each unit. **b** Average Hmax versus average correlation coefficient, ρ for each unit. **c** Same as **b** but for Hmean. The linear trendline, R^2^-, and *p* value are given in each plot. *Black*, *filled circles*, *lines*, and *text* represent slow fill data. *Grey*, *open triangles*, *dotted lines*, and *text* represent isovolumetric data

For slow fill trials, bladder pressure-firing rate relationships with greater Hmax values also had greater Hmean values with R^2^ = 0.33, *p* < 0.01 (Fig. 7a). Interestingly, units that correlated better with bladder pressure had lower Hmax values (R^2^ = 0.13, *p* = 0.01) (Fig. 7b). However, there was no correlation between Hmean and correlation coefficient (R^2^ = 0.01, *p* = 0.48, Fig. 7c). Similar trends were observed with hysteresis values from isovolumetric trials (Hmax vs Hmean: R^2^ = 0.52, *p* < 0.01; ρ vs Hmax: R^2^ = 0.41, *p* < 0.01, ρ vs Hmean: R^2^ = 0.03, *p* = 0.37, Fig. 7).

**Fig. 8:**
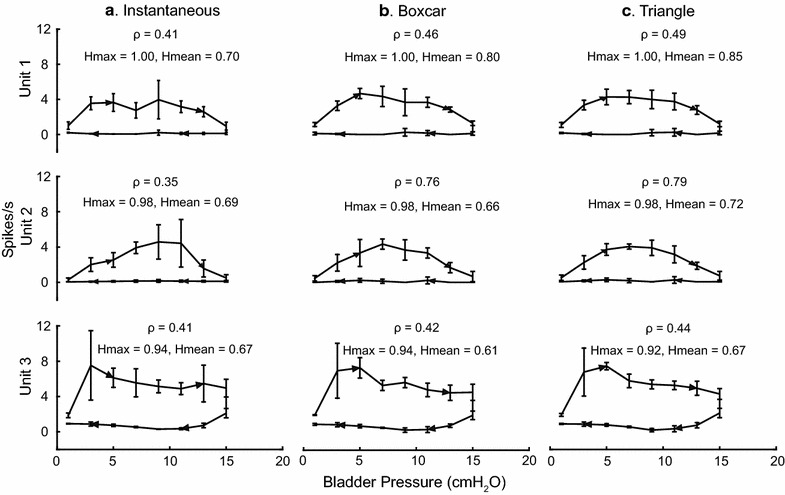
Firing rate analysis comparison. Bladder pressure-firing rate plots averaged over 3 contractions at 0–25 % leak volume showing hysteresis for three different units from experiment 5. Three different methods were used for calculating the firing rate: instantaneous (**a**), boxcar (**b**), and triangle (**c**). Correlation coefficient (ρ), Hmax, and Hmean given for each unit and firing rate calculation method. *Error bars* represent standard deviation

The effect of different firing rate calculation approaches was analyzed. In addition to the non-causal linear filter with triangle kernel (described in “Methods”), two other methods were used to calculate firing rates: instantaneous firing rate and boxcar. At every 0.5 s, the instantaneous firing rate was determined by calculating the reciprocal of the interval between the two spikes surrounding the time point [34]. For the boxcar method (rate histogram) the spike rate at every 0.5 s was calculated as the spike count within 1 s bins divided by the bin size [11, 14]. The correlation coefficient, Hmax, and Hmean were compared for all units for each firing rate approach for all slow fill trials. Overall, little difference was seen in the hysteresis values obtained using the different firing rate methods. Figure 8 shows an example from one experiment comparing the hysteresis values and correlation coefficients for 3 units. Across all experiments, the triangle kernel method resulted in the highest ρ between firing rate and pressure for a given unit and was the method selected for our analysis.

### Comparison to published literature

The hysteresis indices, Hmax and Hmean were calculated for bladder pressure-firing rate relationships from published literature (Table 1). In general, even though the data in these papers were collected under a variety of conditions and from different types of bladder neural signals and sources, similar Hmax and Hmean values were obtained, with Hmax = 0.64 ± 0.13 and Hmean = 0.39 ± 0.14.

**Table 1:** Quantification of hysteresis from bladder pressure/tension-bladder afferent plots in published literature

References	Figure # and trace #	Animal model	Neural source and signal type	Bladder volume	Test state	Hmax	Hmean
Bruns [23]	5—unit 3 *ρ*: 0.71	Cat	S1 and S2 DRG	20 ml	None, bladder at isovolumetric state	0.65	0.43
	5—unit 4 *ρ*: 0.68	Cat	S1 and S2 DRG	20 ml	None, bladder at isovolumetric state	0.87	0.71
Downie [8]^#^	7A^++^	Cat	Pelvic nerve filament	NR	Distension/withdrawal cycles under volume-step conditions	0.57	0.25
	7D	Cat	Pelvic nerve filament	NR	Distension/withdrawal cycles under pressure-step conditions	0.48	0.20
Jezernik et al. [35]	7—1st cycle	Pig	Pelvic ENG	Empty	60 ml bolus injection/withdrawal at 116 ml/min fill rate	0.52	0.34
	7—2nd cycle	Pig	Pelvic ENG	Empty	60 ml bolus injection/withdrawal at 133 ml/min fill rate	0.77	0.49
Winter et al. [16]	2—unit 1	Cat	Detrusor afferent	Up to 10 ml	5 ml bolus injection/withdrawal	0.72	0.31
	2—unit 2	Cat	Detrusor afferent	Up to 10 ml	5 ml bolus injection/withdrawal	0.71	0.35
	2—unit 3	Cat	Detrusor afferent	Up to 10 ml	5 ml bolus injection/withdrawal	0.60	0.35
	2—unit 4	Cat	Detrusor afferent	Up to 10 ml	5 ml bolus injection/withdrawal	0.68	0.40

*NR* not reported

Hmax and Hmean values are average values (across multiple loops per unit) for Ref. [16] and [23]

^#^ Firing rate versus tension on bladder surface, not pressure

^++^ The hysteresis relationship was opposite in direction than normal

## Discussion

The goal of this study was to quantify the hysteresis observed in bladder pressure-bladder afferent relationships. A total of 66 bladder afferents across S1 and S2 DRG in 5 cats were identified that correlated with bladder pressure and bladder contractions. Hysteresis was observed in the firing rate responses of these afferents to bladder pressure. Analysis of contractions during slow fill and isovolumetric trials yielded Hmax values ranging from 0.38 to 1.00 and Hmean values ranging from 0.09 to 0.76 (Fig. 4). Hysteresis values did not differ across different volume ranges (Figs. 5, 6).

In general, afferents that correlated better with bladder pressure tended to have lower Hmax values (Fig. 7b). However, there was no relationship observed between Hmean and how well an afferent correlated with bladder pressure (Fig. 7c). On closer examination, the inverse relationship seen between Hmax and correlation coefficient for the slow fill trials was observed in two of the five animals (experiments 2 and 3). For two other animals (experiments 1 and 5), this was actually a positive relationship. An inverse relationship between Hmax and correlation coefficient was also observed in both experiments (3 and 4) for the isovolumetric trials. Intuitively, it would seem to follow that there would be less hysteresis where there is a more linear firing rate-pressure relationship, but this was not the case for all animals. These individual differences in responses may partly be explained by how Hmax is calculated. Hmax is determined by only the maximum difference in firing rate, compared to Hmean which gives the average difference in firing rate over the whole loop. Hence, Hmax is more susceptible to outlier firing rate differences over a cycle as it simply gives the largest relative firing rate difference. Hmean, on the other hand, is the mean relative firing rate difference over the pressure range and provides a more representative measure of the hysteresis over all the cycles and seems to be less affected by large variability in firing rate over a cycle and from one cycle to the next (Fig. 2c). Hmean in general is lower if there is more variability in the amount of hysteresis across individual loops and if there is less hysteresis. Furthermore, if both Hmax and Hmean are large and the ratio of Hmax/Hmean is close to 1, this in general reflects more hysteresis in the system and larger, more uniform loops. Higher ratios of Hmax/Hmean indicate more peculiar loops [32] and more variability in the loops between cycles.

Several factors may have contributed to variability in hysteresis loops (e.g. Fig. 2c ii) beyond natural irregular action potential firing, including inconsistencies in spike isolation and sorting and the firing rate calculation. We sought to minimize these sources of variability through several approaches. We utilized consistent unit thresholding and a fixed cutoff for determining bladder units (correlation coefficient ≥ 0.2). All sorted bladder units were manually verified by the same person. We also performed a preliminary hysteresis analysis comparing different firing rate calculations. We did not see a difference in computed hysteresis values between different methods (Fig. 8), and proceeded with a consistent firing rate calculation. Nevertheless, we cannot rule out potential contributions of these and other sources of error in the calculated hysteresis values. Still bladder afferent units had higher Hmax and Hmean values (on average across both data sets higher than 0.7 and 0.4, respectively), particularly when compared to non-bladder units which generally had a very small Hmean (close to 0).

We applied the same equations for calculating Hmax and Hmean to neural activity-bladder pressure (or tension) plots in published literature (Table 1). The range of Hmax and Hmean values from those plots were comparable to those values calculated from our data (Figs. 4, 5, and 6). Note that the data in Table 1 was collected from a variety of animal models (cat, pig, and rat), fill rates, and signal sources (dorsal root ganglia and pelvic nerves). An advantage of the hysteresis calculation method used in this study is that it does not depend on the actual units of the input–output signals. Further testing could be done to rigorously compare effects of different types of signals and/or signal sources and different fill rates (for example, comparing rapid injection of fluid versus more physiological fill rates).

Though our results were generally consistent, there was variability in the data sets. Some slow fill trials had many contractions (up to 16) whereas others had only 3 contractions. Thus we chose a set number of contractions per volume range. In addition, not all volume ranges within a fill cycle met our criteria for contraction count and size. Therefore, all volume ranges were not represented in all trials and animals. Some animals also had more bladder units than others. Another limitation of this study was the small sample size for the isovolumetric data (only 2 animals), though the results did overlap with the values from the slow fill trials. Another potential confounding effect was the difference in the infused saline temperature for experiment 5 compared to the other experiments. In experiment 5, saline was infused into the bladder at body temperature compared to room temperature for the other experiments. Room temperature saline infusion is a standard procedure in bladder studies [6, 14]; however, functional differences can occur depending on the temperature of the saline [36]. We did see a higher average Hmean value for the “warmer” infusion compared to 3 out of the other 4 “cooler” infusions which may be due to this difference in temperature, though there was no difference observed for Hmax.

The origins of the pressure-firing rate hysteresis could be both myogenic and neurogenic. This may be due to the intrinsic mechanical properties of the bladder [37]. The bladder wall consists of smooth muscle, elastin, and collagen and has non-linear elastic, viscous, and plastic properties [38, 39]. With repeated filling and emptying of the bladder, length-tension curves of the bladder produces characteristic hysteresis loops [40]. Even in the absence of neural innervation, outside of the body, the bladder muscle displays this hysteretic property, further demonstrating that hysteresis is inherent in the muscle. Subjecting bladder muscle strips to sequential stretching and relaxing resulted in shifts of the tension-time and length-tension curves in a manner typical for materials with hysteresis [41]. This non-linear behavior is also demonstrated in stress–strain curves after cyclic loading of the bladder muscle [38, 42].

This hysteretic relationship may not only be explained by myogenic factors. Neuronal mechanisms may also play a role, though the particulars of this are not clear. Hysteresis could result from the membrane properties of the afferent fibers themselves and the mechanosensitive receptors. The primary mechanosensitive receptor in the bladder is thought to be the transient receptor potential vanilloid 4 (TRPV4) channel [43]. It is not known if the TRPV4 receptors demonstrates hysteresis, though another family member, the TRPV3, a temperature-sensitive receptor [44], has been shown to demonstrate hysteresis. Furthermore, other mechanosensory systems such as muscle spindles and joint receptors in cats have been reported to demonstrate hysteresis [21, 45]. Many mechanoreceptors have also been reported to show adaptation with decreases in frequency and/or amplitude with increasing input stimuli which may be attributed to the viscoelastic nature of the receptors [46] and some bladder afferent responses may also demonstrate adaptation [47]. However, more detailed studies would be required to confirm neurogenic causes of this non-linear behavior or if it was solely due to the biomechanics of the muscle itself, which is beyond the scope of this study.

The rate of bladder filling has been reported to affect the bladder’s intrinsic mechanical properties, with plastic behavior dominating during natural fill rates and viscous forces coming into play during rapid filling. Bladder compliance also decreases with increasing fill rates [48]. We infused saline at a near-physiological rate, in the medium–high range [31], so both plastic and viscous forces likely contributed to our results. Additionally afferent activity has been reported to be lower for a given pressure at higher fill rates (compared to more natural fill rates) [9]. Even though this may have occurred, the hysteretic relationship is still evident and the amount of hysteresis we see is comparable to values calculated from previous studies. Those studies used different fill rates and one of them, specifically looked at tension versus afferent activity (Table 1), whose relationship is reported to be independent of fill rates [9]. Ultimately, our goal is to develop a real-time closed-loop neuroprosthesis to determine bladder pressure from neural activity; hence, we are focusing on relationship between bladder pressure and bladder afferent activity.

Given the hysteretic relationship between bladder afferent activity and bladder pressure, a simple linear equation does not adequately describe the relationship between these two. There are closed-loop bladder control approaches that do not take into account hysteresis, which have demonstrated minimal success so far. For example, some studies have focused on estimated bladder volume instead of pressure [49–51], though pressure may be a more reliable estimator than volume [52]. Another study has shown a proof of concept closed-loop model using direct pressure monitoring as the feedback signal [53], but this requires a reliable way to directly measure bladder pressure that comes with its own challenges [54]. A closed-loop model for bladder control that takes into account hysteresis may result in a more realistic estimation of bladder pressure from afferent activity than using a simple linear regression model [23]. One approach could be using piecewise polynomials to model tonic increase in bladder pressure and contractions, and to use estimated hysteresis to adjust for the changing pressure-firing rate relationship on the rising and falling edges of contractions. Another method could be incorporating derivatives to model the hysteresis loop, similar to models proposed for dynamic hysteresis loops [55]. As we did not see differences in hysteresis values across different volume ranges, one hysteretic value may be applicable at different bladder volumes. Further experiments and algorithm development are needed to determine an optimal approach, which will also need to account for the effects of movement that will occur in a behaving implant recipient.

## Conclusions

We did a quantitative assessment of the hysteretic relationship between bladder pressure and afferent activity, which has been previously commented on but not described quantitatively. The amount of hysteresis is similar across slow fill and isovolumetric trials and at different volumes and is comparable to values we calculated from published examples. These results provide more information on the relationship between bladder pressure and afferent activity and could be utilized in a closed-loop model for a bladder neuroprosthesis.

## Abbreviations

DRG: dorsal root ganglion (or ganglia)
LUT: lower urinary tract
S1: sacral level 1
S2: sacral level 2
FR: firing rate
P: pressure
Hmax: maximum hysteresis ratio between the firing rate difference at each pressure and the overall firing rate range
Hmean: mean hysteresis ratio between the firing rate difference at each pressure and the overall firing rate range

## References

[1] de Groat WC, Griffiths D, Yoshimura N (2015). Neural control of the lower urinary tract. Compr Physiol..

[2] de Groat WC (1986). Spinal cord projections and neuropeptides in visceral afferent neurons. Prog Brain Res.

[3] de Groat WC (2006). Integrative control of the lower urinary tract: preclinical perspective. Br J Pharmacol.

[4] Vodusek DB (2004). Anatomy and neurocontrol of the pelvic floor. Digestion.

[5] de Groat WC, Nadelhaft I, Milne RJ, Booth AM, Morgan C, Thor K (1981). Organization of the sacral parasympathetic reflex pathways to the urinary bladder and large intestine. J Auton Nerv Syst.

[6] Häbler HJ, Jänig W, Koltzenburg M (1993). Myelinated primary afferents of the sacral spinal cord responding to slow filling and distension of the cat urinary bladder. J Physiol.

[7] Evans JP (1936). Observations on the nerves of supply to the bladder and urethra of the cat, with a study of their action potentials. J Physiol.

[8] Downie JW, Armour JA (1992). Mechanoreceptor afferent activity compared with receptor field dimensions and pressure changes in feline urinary bladder. Can J Physiol Pharmacol.

[9] Satchell P, Vaughan C (1994). Bladder wall tension and mechanoreceptor discharge. Pflugers Arch Eur J Physiol.

[10] Bahns E, Halsband U, Jänig W (1987). Responses of sacral visceral afferents from the lower urinary tract, colon and anus to mechanical stimulation. Pflugers Arch.

[11] Sengupta JN, Gebhart GF (1994). Mechanosensitive properties of pelvic nerve afferent fibers innervating the urinary bladder of the rat. J Neurophysiol.

[12] Iggo A (1955). Tension receptors in the stomach and the urinary bladder. J Physiol.

[13] Morrison J (1997). The physiological mechanisms involved in bladder emptying. Scand J Urol Nephrol.

[14] Shea VK, Cai R, Crepps B, Mason JL, Perl ER (2000). Sensory fibers of the pelvic nerve innervating the rat’s urinary bladder. J Neurophysiol.

[15] Häbler HJ, Jänig W, Koltzenburg M (1990). Activation of unmyelinated afferent fibres by mechanical stimuli and inflammation of the urinary bladder in the cat. J Physiol.

[16] Winter DL (1971). Receptor characteristics and conduction velocities in bladder afferents. J Psychiatr Res.

[17] Talaat M (1937). Afferent impulses in tide nerves supplying the urinary bladder. J Physiol.

[18] Bruns TM, Gaunt RA, Weber DJ (2011). Multielectrode array recordings of bladder and perineal primary afferent activity from the sacral dorsal root ganglia. J Neural Eng.

[19] Hatsopoulos NG, Burrows M, Laurent G (1995). Hysteresis reduction in proprioception using presynaptic shunting inhibition. J Neurophysiol.

[20] Segundo JP, Diez Martínez O (1985). Dynamic and static hysteresis in crayfish stretch receptors. Biol Cybern.

[21] Kostyukov AI, Cherkassky VL (1992). Movement-dependent after-effects in the firing of the spindle endings from the de-efferented muscles of the cat hindlimb. Neuroscience.

[22] Damaser MS (1999). Whole bladder mechanics during filling. Scand J Urol Nephrol Suppl.

[23] Bruns TM, Gaunt RA, Weber DJ (2011). Estimating bladder pressure from sacral dorsal root ganglia recordings. Conf Proc IEEE Eng Med Biol Soc..

[24] Aoyagi Y, Stein RB, Branner A, Pearson KG, Normann RA (2003). Capabilities of a penetrating microelectrode array for recording single units in dorsal root ganglia of the cat. J Neurosci Methods.

[25] Snellings AE, Yoo PB, Grill WM (2012). Urethral flow-responsive afferents in the cat sacral dorsal root ganglia. Neurosci Lett.

[26] Stein RB, Weber DJ, Aoyagi Y, Prochazka A, Wagenaar JBM, Shoham S, Normann RA (2004). Coding of position by simultaneously recorded sensory neurones in the cat dorsal root ganglion. J Physiol.

[27] Weber DJ, Stein RB, Everaert DG, Prochazka A (2007). Limb-state feedback from ensembles of simultaneously recorded dorsal root ganglion neurons. J Neural Eng.

[28] Branner A, Normann RA (2000). A multielectrode array for intrafascicular recording and stimulation in sciatic nerve of cats. Brain Res Bull.

[29] Clark GA, Ledbetter NM, Warren DJ, Harrison RR (2011). Recording sensory and motor information from peripheral nerves with Utah Slanted Electrode Arrays. Conf Proc Annu Int Conf IEEE Eng Med Biol Soc..

[30] Mathews KS, Wark HAC, Warren DJ, Christensen MB, Nolta NF, Cartwright PC, Normann RA (2014). Acute monitoring of genitourinary function using intrafascicular electrodes: selective pudendal nerve activity corresponding to bladder filling, bladder fullness, and genital stimulation. Urology..

[31] Klevmark B (1999). Natural pressure-volume curves and conventional cystometry. Scand J Urol Nephrol Suppl.

[32] Kosmulski M, Próchniak P, Saneluta C (2009). Quantitative assessment of hysteresis in voltammetric curves of electrochemical capacitors. Adsorption..

[33] Winter B. Linear models and linear mixed effects models in R with linguistic applications. 2013. arXiv:1308.5499.

[34] Stein RB, Weber DJ (2004). Editing trains of action potentials from multi-electrode arrays. J Neurosci Methods.

[35] Jezernik S, Wen JG, Rijkhoff NJ, Djurhuus JC, Sinkjaer T (2000). Analysis of bladder related nerve cuff electrode recordings from preganglionic pelvic nerve and sacral roots in pigs. J Urol.

[36] Sugaya K, de Groat WC (2000). Influence of temperature on activity of the isolated whole bladder preparation of neonatal and adult rats. Am J Physiol Integr Comp Physiol..

[37] Danziger ZC, Grill WM (2015). Sensory and circuit mechanisms mediating lower urinary tract reflexes. Auton Neurosci..

[38] Alexander RS (1973). Viscoplasticity of smooth muscle of urinary bladder. Am J Physiol.

[39] Coolsaet BL, van Duyl WA, van Mastrigt R, van der Zwart A (1975). Visco-elastic properties of the bladder wall. Urol Int.

[40] Remington J, Alexander R (1955). Stretch behavior of the bladder as an approach to vascular distensibility. Am J Physiol.

[41] Finkbeiner AE (1999). In vitro responses of detrusor smooth muscle to stretch and relaxation. Scand J Urol Nephrol.

[42] Rubod C, Brieu M, Cosson M, Rivaux G, Clay J-C, de Landsheere L, Gabriel B (2012). Biomechanical properties of human pelvic organs. Urology..

[43] Mochizuki T, Sokabe T, Araki I, Fujishita K, Shibasaki K, Uchida K, Naruse K, Koizumi S, Takeda M, Tominaga M (2009). The TRPV4 cation channel mediates stretch-evoked Ca2 + influx and ATP release in primary urothelial cell cultures. J Biol Chem.

[44] Xu H, Ramsey IS, Kotecha SA, Moran MM, Chong JA, Lawson D, Ge P, Lilly J, Silos-Santiago I, Xie Y, DiStefano PS, Curtis R, Clapham DE (2002). TRPV3 is a calcium-permeable temperature-sensitive cation channel. Nature.

[45] Lennerstrand G (1968). Position and velocity sensitivity of muscle spindles in the cat. I. Primary and secondary endings deprived of fusimotor activation. Acta Physiol Scand.

[46] French A (1984). The receptor potential and adaptation in the cockroach tactile spine. J Neurosci.

[47] Iijima K, Igawa Y, Wyndaele J-J, De Wachter S (2009). Mechanosensitive primary bladder afferent activity in rats with and without spinal cord transection. J Urol.

[48] Vaughan C (1995). Urine storage mechanisms. Prog Neurobiol.

[49] Mendez A, Sawan M, Minagawa T, Wyndaele JJ (2013). Estimation of bladder volume from afferent neural activity. IEEE Trans Neural Syst Rehabil Eng.

[50] Park JH, Kim CE, Shin J, Im C, Koh CS, Seo IS, Kim SJ, Shin HC (2013). Detecting bladder fullness through the ensemble activity patterns of the spinal cord unit population in a somatovisceral convergence environment. J Neural Eng.

[51] Saleh A, Sawan M, Elzayat EA, Corcos J, Elhilali MM (2008). Detection of the bladder volume from the neural afferent activities in dogs: experimental results. Neurol Res.

[52] Choudhary M, van Asselt E, van Mastrigt R, Clavica F (2015). Neurophysiological modeling of bladder afferent activity in the rat overactive bladder model. J Physiol Sci..

[53] Lin Y, Lai C, Kuo T, Chen C, Chen Y, Young A, Chen S, Lai JS, Hsieh T, Peng C (2014). Dual-channel neuromodulation of pudendal nerve with closed-loop control strategy to improve bladder functions. J Med Biol Eng..

[54] Gaunt RA, Prochazka A (2006). Control of urinary bladder function with devices: successes and failures. Prog Brain Res.

[55] Chua LO, Stromsmoe KA (1971). Mathematical model for dynamic hysteresis loops. Int J Eng Sci.

[56] Bruns T, Ross S, Khandwala N, Mahar C, Sperry Z. Bladder neuron hysteresis. Open Science Framework. 2016. 10.17605/OSF.IO/2Y4DP.

[57] Bruns TM, Weber DJ, Gaunt RA (2015). Microstimulation of afferents in the sacral dorsal root ganglia can evoke reflex bladder activity. Neurourol Urodyn.

